# Diseases of the Nervous System

**Published:** 1906-04-14

**Authors:** 


### Progress in Medicine and Surgery.

### DISEASES OF THE NERVOUS SYSTEM.

*Continued from Vol. XXXIX., page 442.*

**Some Spinal Cord Degenerations.**—Lesions of the posterior columns of the cord are not confined to locomotor ataxia. Secondary degenerations occur, ascending and descending, as a result of transverse lesions of the cord from various causes, and ascending tracts of degeneration in the posterior columns follow lesions of the posterior nerve-roots whether traumatic or non-traumatic. But changes in the posterior columns other than secondary and similar to those found in tabes dorsalis have been observed in several diseases, and these have been described by R. T. Williamson,[18] who divides them into two important groups:—(1) Changes in the posterior columns which commence in the posterior root fibres directly these fibres pass through the spinal pia mater into the cord; (2) changes in the posterior columns which do not commence at the point just mentioned. Group (1) is concerned with the intramedullary course of the posterior root fibres. It has been shown that at the point where these pass through the pia mater they lose not only their external sheath, but for a very short distance their medullary sheath also. The latter is, however, resumed directly the fibres emerge on the inner side of the pia mater. The point where the myelin sheath is lost and the axis cylinders are consequently for a very short distance naked, has been termed the vulnerable point, and in several diseases is the seat of origin of degenerative changes which are continued in the intramedullary portions of the fibres. Such a degeneration is seen in early cases of locomotor ataxia. It is, however, maintained by some, as by Nageotte, that the changes start as the fibres pierce the spinal dura mater, but many cases have been recorded in which the fibres have appeared normal just outside and degenerated just inside the pia mater. In severe cases of diabetes mellitus Williamson has found intramedullary root fibre changes similar to those noted in tabes, usually confined to the inner portion of the intramedullary fibres, and not affecting the external bundle of fibres which passes to Lissauer's zone. In this connection it is interesting to recall that in severe cases of diabetes symptoms similar to some of those found in tabes may occasionally be noted, such as abolition of deep reflexes, perforating ulcer, and pains in the legs occasionally of a stabbing nature. Rarely also this writer has observed abolition of the vibrating bone sensation, similar to the loss observed in some early cases of tabes dorsalis in which other forms of sensation are normal. These nerve root changes have also been observed in intracranial tumour, and have been here ascribed by Batten and Collier to traction on the roots secondary to distension of the arachnoid. They have been observed in tumour of the third ventricle and in cerebellar tumour. The involvement in all these cases of the intramedullary sensory fibres at the same spot—namely, immediately they have passed through the pia mater, is considered very suggestive of the action of toxic lymph or blood on the naked axis cylinders in this region. The degeneration has also been observed in general paralysis, sarcoma of the spinal dura mater, chronic ergotism, carcinoma of various organs, peripheral neuritis, and Friedreich's disease. In group (2) the intramedullary fibres are not degenerated immediately after passing through the pia mater. The changes are variable. Degenerations in the posterior and lateral columns have been found in pellagra, and in some cases of severe anæmia, also in ataxic paraplegia, Erb's syphilitic spinal paralysis, syringo-myelia, the peroneal type of muscular atrophy, senile paraplegia, and central progressive muscular atrophy. Accompanying degeneration in the posterior root zone or extramedullary root fibres is often absent, but in several of these conditions other associated cord lesions are present. The changes described in the intramedullary portions of the sensory roots by Williamson as occurring in tabes are quite in accord with those stated to exist in general paralysis by Orr and Rows.[19] They find that in general paralysis the external division of the intramedullary roots, the extramedullary roots, and Lissauer's zone are unaffected, but that the internal divisions of the sensory roots after their entrance into the cord show degenerative changes most marked in the lumbar region, and diminishing progressively as the dorsal and cervical portions are reached. These changes they consider almost absolutely coincide with the sclerotic areas found in early cases of tabes dorsalis. Rejecting the various hypotheses which have been advanced in explanation of this sclerosis, such as primary ganglionic degeneration, Wallerian degeneration secondary to neuritis, parenchymatous degeneration of the roots, meningeal compression, etc., the authors conclude that the intramedullary portions of the sensory roots are rendered more vulnerable by the loss of their neurilemma, which exercises a protective influence probably more vital than mechanical, that they are therefore rendered more susceptible to the action of circulating toxins, and that therefore tabes is a system disease beginning "as a parenchymatous degeneration of the sensory protoneurons starting at the point where the neurilemma is lost," and presumably due to some toxin circulating in the blood or lymph. On the clinical side the variety of symptoms met with in tabes is well illustrated by three patients, who formed the subject of a recent lecture by C. O. Hawthorne.[20] The first case, a man of 35, otherwise healthy, had suffered for four years from intermittent pains, chiefly in the legs and feet, described as like "a deep toothache," that is not like "lightning pains." He had been treated as suffering from rheumatism, gout, or neuralgia. No ataxy was present, but routine examination of the nervous system revealed a measure of Argyll-Robertson pupil and total loss of knee and heel jerks, thus showing central nervous degenerative changes. From the patient's point of view the sole evidence of disease here was pain, and he could almost be classed among the cases of pure "tabetic neuralgia" described by Gowers, in which pain may be the only subjective or objective symptom for years. The second patient, a man aged 54, complained of loss of sight in one eye, which he could prove to be of recent origin. Examination revealed a unilateral optic atrophy. The other fundus was normal and vision good. There was no other complaint, and such a case might have been put down to embolism or thrombosis of the retinal artery or to retro-bulbar neuritis. But examination revealed a greatly restricted visual field in the apparently sound eye—showing some measure of optic atrophy here also, Argyll-Robertson pupil, absent knee and ankle jerks, and a narrow zone of anæsthesia encircling the trunk in the region of the nipple. The third man, aged 44, complained merely of double vision, and careful examination could only detect paralysis of the left third nerve. Inquiry, however, elicited a history of syphilis and a previous paralysis of the sixth nerve. Full doses of iodide of potassium produced no effect after three weeks, as might have been expected had the lesion been due to gumma or syphilitic meningitis. The first patient was improving on chloride of aluminium, hot-water douches nightly to the spine, with a spinal mustard pack once a week, and ten grains of acetanilide with half an hour's rest on the occurrence of pain. J. Collins[21] states that in upwards of three hundred cases of tabes of which he has records, in two-thirds the diagnosis had been missed and the patients had been under treatment, sometimes for years, for rheumatism, flat feet, nervous prostration, etc., so that there seems some reason for his plea that general practitioners should pay more attention to systematic examination of the nervous system. E. F. Trevelyan [22] has recorded a case of family tabes occurring in husband, wife, and daughter. The daughter showed no sign of inherited syphilis, but there was fairly strong indirect evidence of its presence in the parents. Giannelli [23] has recorded a case of juvenile tabes commencing in a girl when 17 years of age. A case of juvenile general paralysis has also been placed on record by P. C. Knapp.[24] There was a very clear history of inherited syphilis. The patient, a boy of 13 years, had shown some change of disposition since an emotional shock two years previously, but had been worse for six months. There were noted irritability and violence of temper, amnesia for recent events, blurred speech, indistinct handwriting, mental deficiency, absence of deep reflexes, slight papillitis, fæcal incontinence, and some difficulty in swallowing. Headache, convulsions, and tremor were absent.

[18] Med. Chron., Oct. [19] Ibid., Aug., p. 318. [20] Lancet, Sept. 30. [21] Med. Record, Sept. 16. [22] Lancet, Sept. 9. [23] Jour. of Nerv. and Ment. Dis., Aug., p. 543. [24] Ibid., Oct., p. 665.

